# Giloy Ghanvati (*Tinospora cordifolia* (Willd.) Hook. f. and Thomson) Reversed SARS-CoV-2 Viral Spike-Protein Induced Disease Phenotype in the Xenotransplant Model of Humanized Zebrafish

**DOI:** 10.3389/fphar.2021.635510

**Published:** 2021-04-19

**Authors:** Acharya Balkrishna, Lakshmipathi Khandrika, Anurag Varshney

**Affiliations:** ^1^ Drug Discovery and Development Division, Patanjali Research Institute, Haridwar, India; ^2^ Department of Allied and Applied Sciences, University of Patanjali, Patanjali Yog Peeth, Haridwar, India

**Keywords:** tinopsora cordifolia, ayurved, viral disease, SARS–CoV–2, inflamation, zebrafish, xenotranplantation, giloy ghanvati

## Abstract

The current Severe Acute Respiratory Syndrome disease caused by Coronavirus-2 (SARS-CoV-2) has been a serious strain on the healthcare infrastructure mainly due to the lack of a reliable treatment option. Alternate therapies aimed at symptomatic relief are currently prescribed along with artificial ventilation to relieve distress. Traditional medicine in the form of Ayurveda has been used since ancient times as a holistic treatment option rather than targeted therapy. The practice of Ayurveda has several potent herbal alternatives for chronic cough, inflammation, and respiratory distress which are often seen in the SARS-CoV-2 infection. In this study we have used the aqueous extracts of *Tinospora cordifolia* (willd.) Hook. f. and Thomson in the form of Giloy Ghanvati, as a means of treatment to the SARS-CoV-2 spike-protein induced disease phenotype in a humanized zebrafish model. The introduction of spike-protein in the swim bladder transplanted with human lung epithelial cells (A549), caused an infiltration of pro-inflammatory immune cells such as granulocytes and macrophages into the swim bladder. There was also an increased systemic damage as exemplified by renal tissue damage and increased behavioral fever in the disease induction group. These features were reversed in the treatment group, fed with three different dosages of Giloy Ghanvati. The resultant changes in the disease phenotype were comparable to the group that were given the reference compound, Dexamethasone. These findings correlated well with various phyto-compounds detected in the Giloy Ghanvati and their reported roles in the viral disease phenotype amelioration.

## Introduction

Herbal medicine in the form of Ayurveda, is a well-documented and widely accepted traditional medical system in India. The use of traditional and complementary medicine is often seen in chronic illness and a general improvement of wellness (Hankey, 2010; Balkrishna, 2015). During recent times, the use of herbal based formulations has been on the rise in the direct treatment of infectious diseases. WHO has also been encouraging the research and development of traditional medicine in areas that are convergent with the modern medicine to help tackle the unique challenges posed in the 21^st^ century (WHO, 2019).

*Tinospora cordifolia* (willd.) Hook. f. and Thomson is a climbing shrub that is widely distributed throughout the tropical and subtropical regions of Asia, Africa, and Australia. The different members of this family are a rich source of polysaccharides, terpenes, and alkaloids that have demonstrated activity as anti-inflammatory (Tiwari et al., 2014), immune-stimulatory including phagocytosis (Sharma et al., 2012), anti-diabetic (Sharma et al., 2013), anti-oxidant (Imtiyaj Khan et al., 2011) among others (Panchabhai et al., 2008). Compounds belonging to different classes such as Sesquiterpenes, Phenylpropanoids, and alkaloids were isolated and identified from various water and solvent fractions (Kapil and Sharma, 1997; Cho et al., 2001). Though not much work has been done to demonstrate anti-viral activity of this plant, aqueous extract of the stems of *T. cordifolia* was shown to augment the immune protective response of a commercially available Infectious Bursal disease vaccine in chicks (Sachan et al., 2019). In-silico studies using Berberine, Isocolumbin, Magnoflorine, and Tinocordiside showed high binding affinity for the surface glycoprotein, receptor binding domain, RNA dependent RNA polymerase, and main protease of SARS-CoV-2 virus (Sagar and Kumar, 2020).

The SARS-CoV-2 virus modality has been thoroughly investigated and the accumulation of pro-inflammatory cytokines and immune cells in the target tissue has been shown to be the main culprit for large scale systemic disease (Abebe et al., 2020; Inciardi et al., 2020). The robust inflammatory response as a result of viral entry, has been shown to be associated with a greater risk of long term multi-organ damage and adverse outcomes. As of today, there is no effective and recommended therapy for the novel virus; clinical management consists mainly of supportive care and symptomatic relief.

Since, the SARS-CoV-2 infection targets multiple organs, a holistic approach that can alleviate multiple symptoms may be the ideal answer to the current crisis. Ayurveda system of medicine is based on restoring balance to the body rather than just treating the source of the disease. Therapies typically are formulations that include complex herbal mixtures, minerals and metals or their bhasmas (ash, mineral salts treated with herbal concoctions and fried in metallic containers) (Meulenbeld, 1999; Liu et al., 2019). *T. cordifolia* has been prescribed in ayurveda for infective conditions, sporadic fever, skin diseases, urinary disorders, and eye diseases (Chi et al., 2016).

Giloy Ghanvati is the aqueous extracts of *T. cordifolia* that has been manufactured in the form of tablets by Patanjali Ayurveda Limited, Haridwar, India. In the current study, we have used a humanized zebrafish model for determining the effect of Giloy Ghanvati in reversing the inflammation, and tissue damage caused by the induction of SARS-CoV-2 spike protein.

Zebrafish (*Danio rerio*) has been a reliable model for infectious diseases and studying the immune function in response to the introduction of human viruses (Sullivan and Kim, 2008; Van Dycke et al., 2019). The similarities in the immune cells, cytokines, and the presence of various receptors that are required for host-pathogen interactions makes the correlation of zebrafish research to humans, very much relevant (Targen et al., 2020). Xeno-transplantation was achieved by introducing human lung epithelial A549 cells into the swim bladder of the fish (Shen et al., 2020).

We demonstrate the pro-inflammatory changes and tissue damage induced by SARS-CoV-2 spike-protein induction in the zebrafish and show the rescue of the disease pathology upon treatment with Giloy Ghanvati through morphological examination of the swim bladder and kidney, quantitate the immune cell infiltration through cytology, and finally show the restoration of behavioral fever and mortality induced by the spike protein.

## Materials and Methods

### Chemicals and Reagents

Recombinant spike-protein of SARS-CoV-2 virus was procured from Bioss Antibodies (Woburn, MA, United States). Giloy Ghanvati (made of the extracts of *Tinospora cordifolia* (willd.) Hook. f. and Thomson) tablets were sourced from Patanjali Ayurveda Limited (Haridwar, Uttarakhand, India), with batch number AAI20/557 and expiry date of May 2022. All other chemicals and reagents used in the study were at least of an analytical grade and procured from Sigma-Aldrich India Limited (Bengaluru, Karnataka, India).

### HPLC Analysis of Giloy Ghanvati

#### Preparation of Samples

Giloy Ghanvati is available as tablets and for this analysis, the tablets were finely powdered and 0.5 gm powder was diluted with 10 ml methanol: water (70:30) and sonicated for 30 min. The extract was centrifuged at 8000 rpm for 5 min at 4°C and filtered using 0.45 µm nylon filter. This was labeled as a working solution and was used for the analysis of Cordifolioside A, Palmatine and Beta-Ecdysone. The working solution was further diluted to 10 times with same solvent as above and used for the analysis of Magnoflorine.

Standards used for comparative analysis were dissolved in methanol to get the desired concentrations. Palmatine hydrochloride hyrate (Potency-97.0%) and Magnoflorine (Potency-99.0%) were procured from Sigma-Aldrich (St. Louis, MO, United States), Cordifolioside A (Potency-98.6%, ChemFaces, Wuhan, Hubei Province, China), and Beta-Ecdysone (Potency-99.0%, Tokyo Chemical Industries, Tokyo, Japan) were used as standards.

### Zebrafish Husbandry

Standard protocols for zebrafish rearing and maintenance were followed. Fish were bred and reared in a fish rearing facility under guidelines from the Committee for the Purpose of Control and Supervision of Experiments on Animals (CPCSEA), India. Wild-Type *Danio rerio* were maintained in freshwater at a temperature of 27°C (±1°C) at a stocking density of two fish per liter water. The general characteristics of the fish used in the study are listed in Table 1. The tanks were under an automated photoperiod of 14-h light and 10-h dark (Westerfield, 1993; Avdesh et al., 2012; Aleström et al., 2020). Water and housing standards were maintained according to ethical standards and were overseen by the institutional animal ethics committee (vide protocol number: Go062020/IAEC). Feeding was on a 24-h cycle and each fish was given commercially available dry feed (TetraBit, Spectrum Brands Pet LLC, Blacksburg, VA, United States) according to their body weight at 5 mg per Gram.

**TABLE 1:** Inclusion criteria for model zebrafish in the study.

Gender	Male and female
Age	1 year
Body weight	0.5 g
Body length	25–30 mm
No. of fish per group	24
No. of fish per tank	12
Tank capacity	6 L

### Cell Culture

Human lung alveolar epithelial cells (A549) were procured from ATCC (Licensed repository in India at National Center for Cell Sciences, NCCS, Pune, Maharashtra, India). Cells were cultured in Dulbecco’s Modified Eagle’s Medium (DMEM, High Glucose) supplemented with 10% (v/v) Fetal Bovine Serum and 1% (v/v) Penicillin-Streptomycin solution (Invitrogen Life Technologies, Carlsbad, CA, United States). Stock cells frozen under liquid nitrogen were passaged at least twice before using for xeno-transplantation. Culture at about 70% confluent and at the third passage was used in the study.

### Anesthesia for Xeno-Transplantation and Disease Induction

Water at a temperature lower than that is used for regular zebrafish maintenance was utilized as a means of anesthesia for the zebrafish (Chen et al., 2014; Oskay et al., 2018). Two different anesthesia tanks were set-up to maintain a constant temperature of 17 and 12°C. Changes to the operculum movement was used as a measure to determine the effect of cold water for anesthetization. The fish from the respective clutches were individually transferred to the 17°C tank and when the operculum movement was reduced visibly, the fish was transferred to the 12°C tank. The lack of a response to caudal fin touch was taken as a confirmation of complete anesthetization of the fish. After this, each fish was transferred to a stage for injection of either the human lung epithelial cells or the SARS-CoV-2 spike-protein for disease induction.

### Humanized Zebrafish Model Preparation

**FIGURE 1:**
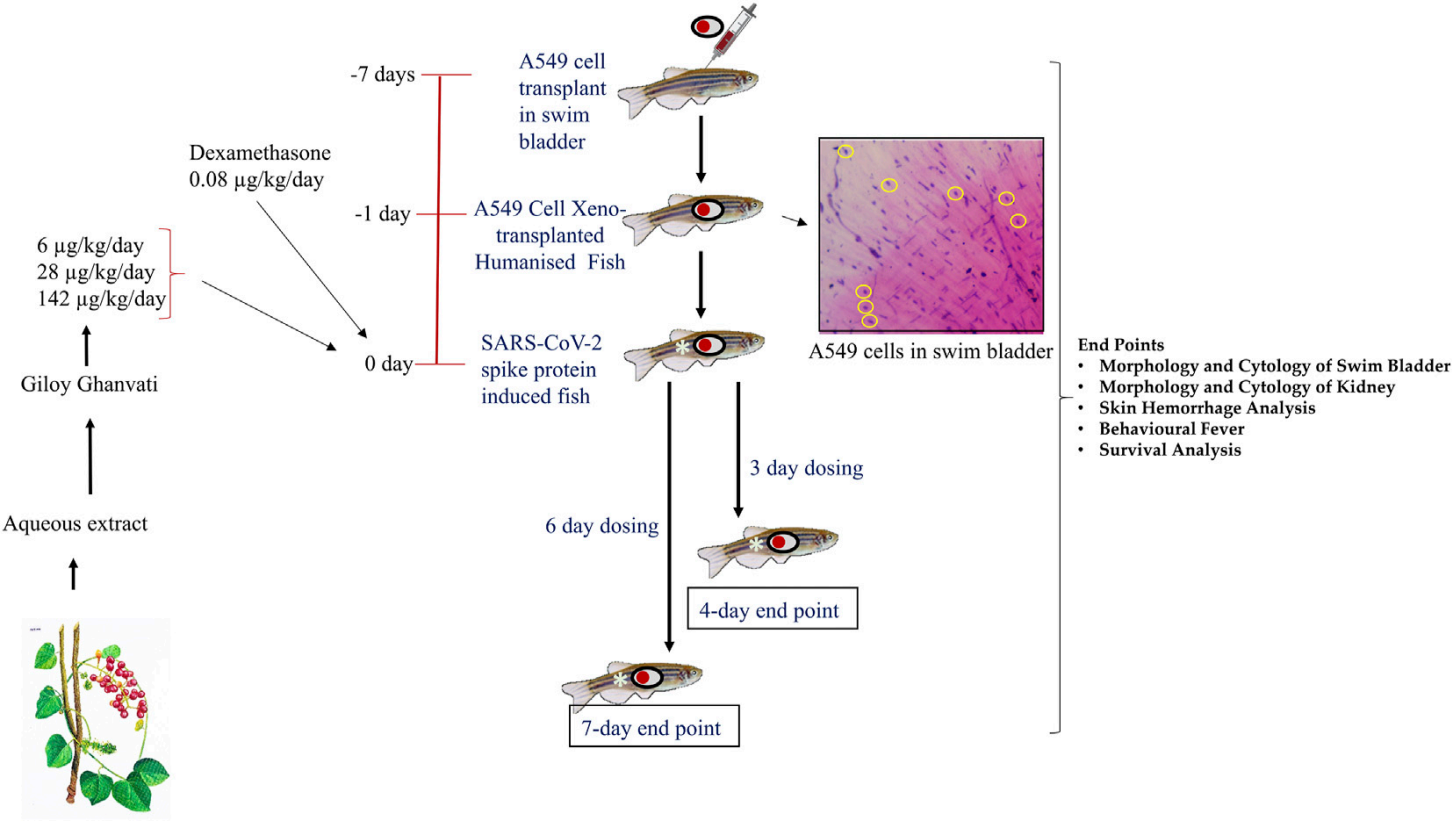
Schematic representing the study design depicting timeline for xenotransplantation, induction with spike-protein of SARS-CoV-2 and treatment with Giloy Ghanvati (GG) and Dexamethasone as a reference drug. The inset shows the hematoxylin-eosin staining of swim bladder cytological smear. The transplanted A549 cells are highlighted in yellow circles.

A549 cells (1 × 10^2^) were resuspended in a minimal volume of PBS at pH 7.4, to be used for generating the zebrafish xenograft model. Cells were injected intramuscularly along the midline at the junction of the trunk and caudal regions of experimental fish. After cell transplantation, the fish were observed for a period of 7 days to ensure that no changes to the physiology of the fish occurred due to the procedure. A few of the fish were sacrificed on the seventh day and the swim bladder was dissected out. Cytological smears of the bladder were stained with Hematoxylin-Eosin to confirm the A549 cell attachment to the swim bladder epithelium of the xeno-transplanted zebrafish (Figure 1). These fish were labeled as the “Humanized Zebrafish” or “HZF” group. Equal number of healthy fish were also injected with the same volume of PBS at pH 7.4 and were labeled as the “Normal control” group (Figure 1).

### Induction of Disease Phenotype using Recombinant Spike-Protein of Sars-Cov-2

Once the A549 cells were successfully transplanted in the zebrafish, SARS-CoV-2 recombinant spike protein was injected into the swim bladder at the site of xenotransplant. Recombinant spike-protein of the SARS-CoV-2 virus was resuspended at a concentration of 1 μg/μL in PBS at pH 7.4. This was further diluted to a working concentration of 1 ng/μL by diluting 1 µL of the stock solution (1 μg/μL) in a final volume of 1 ml. This working solution was used to induce disease phenotype by injecting 2.8 ng (2.8 µL of the 1 ng/μL working solution) into the site of A549 cell xenograft, at the junction of the trunk and caudal regions along the midline. This group was labeled as the “Humanized zebrafish with SARS-CoV-2 Spike protein” or “HZF-SARS-CoV-2-S” (Figure 1).

### Dosing of Zebrafish with Either the Reference Drug or Giloy Ghanvati

Each tablet of Giloy Ghanvati contains 500 mg of Giloy stem extract powder mixed with 78 mg of excipients. Microcrystalline Cellulose (MCC, 30 mg), Magnesium Stearate (1 mg), Erosil (8 mg), Talcum (6 mg), Gum acacia (28 mg), and Hydroxypropyl methylcellulose (HPMC, 5 mg) form the excipients to give a tablet of 578 mg total weight.

Three different doses of Giloy Ghanvati (GG) were used in the study at 0.2X, 1X, and 5X of human equivalent doses. The human dosage of GG was –2 gm/day according to the manufacturer’s license sheet and the translational dosages were calculated following the principles approved by USFDA (Sharma and McNeill, 2009). Dexamethasone was used as a reference drug and this was used at 1X. The translational doses were calculated to be 80 ng/kg/day for study reference drug, Dexamethasone and 6, 28, and 142 μg/kg/day for GG (Figure 1).

Fish were dosed orally with feed infused with the standard and test compounds at the desired dosages. For this, known quantity of compounds for a 2.5 mg pellet was mixed with the commercially available feed and then extruded into pellets. The GG tablets (*T. cordifolia*) were crushed into a fine powder with a mortar and pestle under liquid nitrogen to prevent moisture accumulation. Each fish isolated from the groups and was fed individually with the required number of pellets per day. The normal control group was fed with the unmodified feed under similar conditions.

### End-points for Screening

Two time points were chosen for the dosing, in one part of the study, the fish were dosed for 3 days and terminated on the fourth day. This time point was referred to as 4 days. In another part, the fish were dosed for 6 days and then terminated on the seventh day; this time point was referred to as 7 days (Figure 1). In a parallel experiment, 24 fish in each of the groups, Normal control, HZF, Disease control, Dexamethasone (1 dose) and Giloy Ghanvati (GG) treatments (3 doses) were studied for survival analysis. Mortality, if any, was recorded daily and then plotted by using Kaplan-Meier curve.

#### Assessment of Behavioral Fever

Behavioral fever is a reflection of the internal body temperature in fish. Since zebrafish is an ectotherm and there is no regulation of the body temperature, the fish moves to an environment that matches the body temperature. To determine the changes to behavior and assess behavioral fever, interconnected tanks with each chamber at a different temperature (23, 29, and 37°C) were used. The temperature of each compartment is automatically maintained and the time spent by each fish in the individual temperature chambers is recorded over a period of 180 s. Behavioral fever was assessed at the end of the study period and if the fish spend more time in the higher temperature chamber, it was recorded as the presence of a fever.

#### Euthanization and Dissection

Fish were euthanized using hypothermal shock at 4°C (Matthews and Varga, 2012). A temperature controlled cooling bath was attached to the euthanasia tank and each fish was individually transferred to the tanks. Cessation of vital signs such as operculum movement, heartbeat, and righting of the equilibrium was considered as a successful euthanization.

The fish was transferred to a stage and dissected through a ventral incision in the skin from the lower jaw to the vent. Care was taken to follow all the institutional animal ethics committee’s guidelines for proper dissection protocol. The intestine was removed carefully without disturbing the swim bladder which is connected to the esophagus. The gonads were removed carefully and discarded.

#### Dissection of Swim Bladder for Morphological and Cytological Analysis

The swim bladder is located ventral to the kidneys and is a gas filled chamber with two distinct compartments (Menke et al., 2011). The posterior lobe is connected to the esophagus through a pneumatic duct. After isolation of the intact swim bladder it was immediately transferred to a beaker with PBS at pH 7.4. The bladder was gently washed once and observed under a stereomicroscope at ×1 magnification (Labomed CM4, Labomed Inc., Los Angeles, CA, United States). The images were captured using a camera attachment with the microscope at 14 MP resolution under transmitted light.

Cytology slides were prepared from whole swim bladder necropsy and were stained with Hematoxylin and eosin. Stained sections were imaged at ×20 magnification using Labomed light microscope (Labomed Inc., Los Angeles, CA, United States) and recorded with a camera attached to the microscope.

#### Morphological and Cytological Observation of Kidney

The zebrafish kidney has three morphologically distinct regions called the head, trunk (or saddle), and the tail. It is present ventral to the vertebral column (Menke et al., 2011). For dissecting the kidney, the fish was pinned with its ventral side up to expose the organ attached to the dorsal body wall. The morphological features of the kidney were imaged under transmitted light using a Labomed CM4 stereomicroscope at ×1 magnification.

Hematoxylin-Eosin stained cytological smears were used to determine necrotic and degenerative changes in the kidney. The stained sections were observed at ×20 objective magnification using a Labomed light microscope. Images were captured using a camera attachment to the microscope.

#### Detection of Clinical Signs of Skin Hemorrhage

Euthanized fish were washed once in PBS at pH 7.4, and patted dry with a paper towel. The fish were transferred to a stage and photographed using a Digital Single Lens Reflex camera under reflected light (Nikon D3100, Nikon Corporation, Chiyoda, Japan).

### Data Analysis

All data points are represented as mean with Standard Deviation (S.D.) and displayed graphically using GraphPad Prism (Version 7.04, GraphPad Software Inc., San Diego, CA, United States). All statistical analysis was carried out by Two-Way ANOVA with Tukey’s multiple comparison for determining significance. A *p*-value less than 0.05 was considered to be significant.

## Results

**FIGURE 2:**
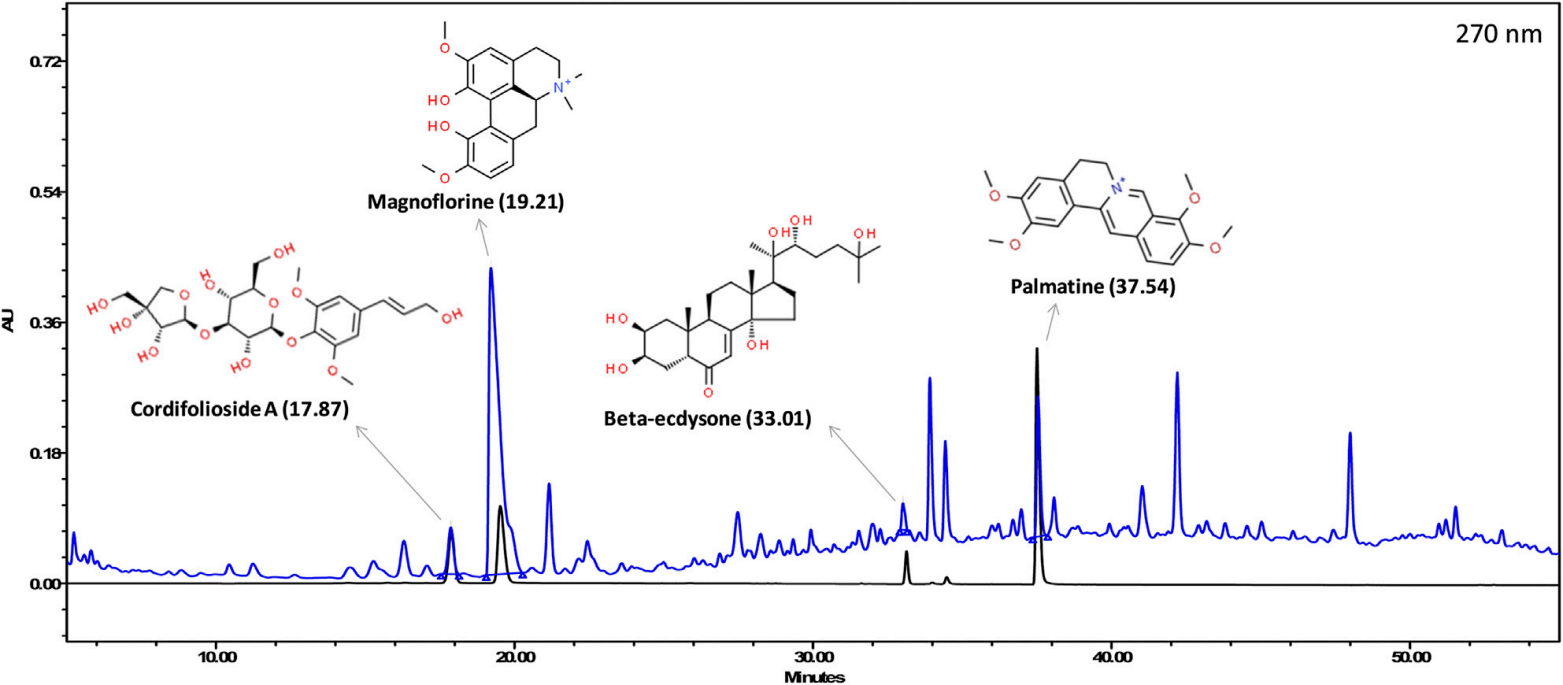
HPLC profile showing an overlap of Giloy Ghanvati aqueous extract (blue chromatogram) and a mixture of standards (black chromatogram). The structures of the phyto-compounds and their retention time are depicted.

The various phyto-compounds of Giloy Ghanvati (GG) were analyzed using a Shodex C18-4E column as described in the methods section. Four constituents were identified based on the retention time and comparison with appropriate standards (Figure 2). Cordifolioside A (1.62 μg/mg dry weight of GG), Magnoflorine (11.00 μg/mg), *β*-Ecdysone (1.75 μg/mg), and Palmatine (1.08 μg/mg) were the main phyto-compounds detected, whereas any others present were below the detection limits. The concentration of Magnoflorine in the GG samples was higher, therefore its peak on the HPLC chromatogram appeared broader, and possibly merged with the peak of another unidentified compound.

### Giloy Ghanvati Reversed SARS-CoV-2 Spike-Protein Induced Edema and Inflammatory Cell Infiltration in Swim Bladder

The swim bladder is a two lobed buoyancy organ which is filled with air when the fish takes a gulp of air through the mouth. The lobes are connected by a wide ductus communicans and they pulsate rhythmically. The inner gas gland is enveloped by a mesothelial tunica externa and is made up of epithelial cells, a smooth muscle layer, and a submucosal layer where most of the vasculature resides.

**FIGURE 3:**
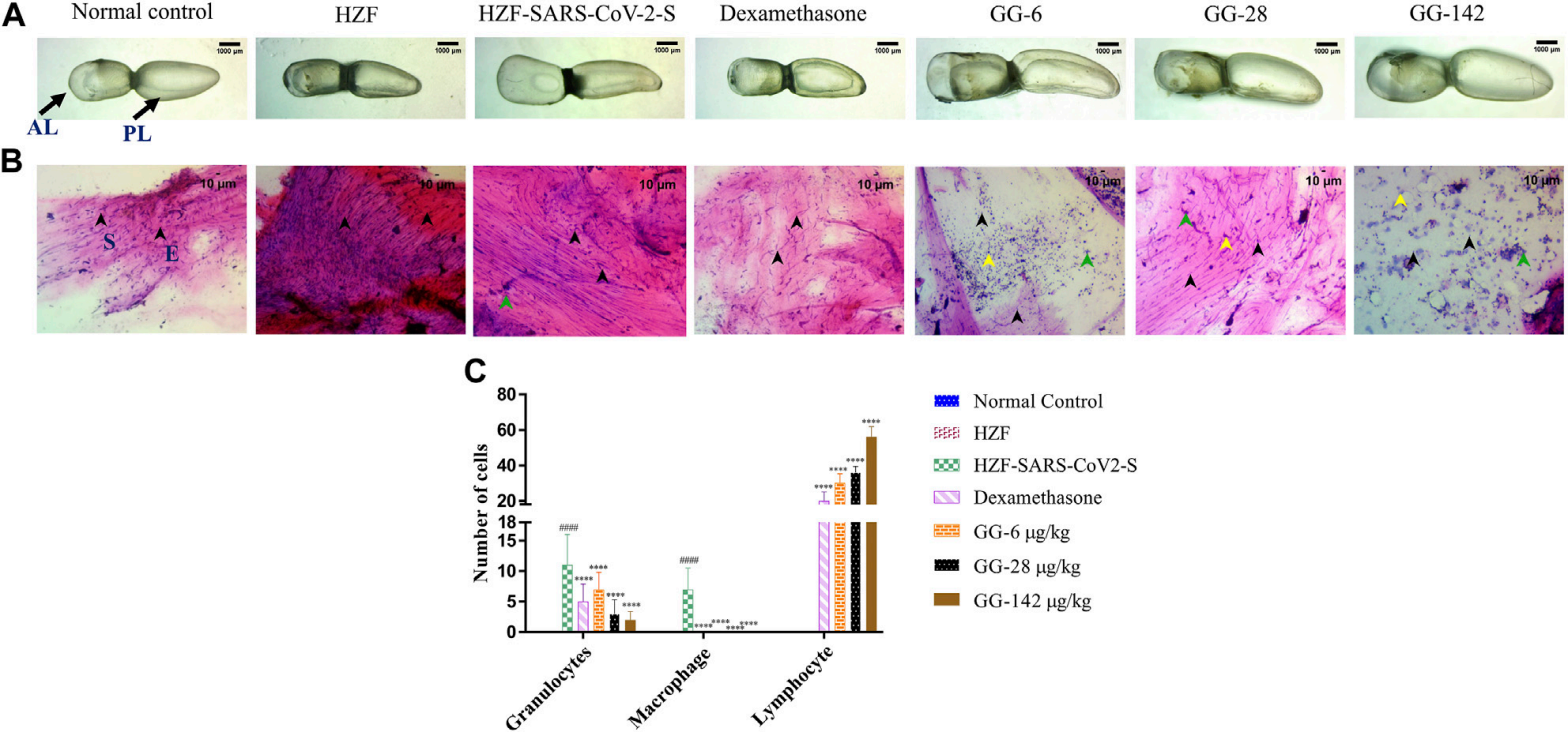
4-days time point to determine changes in morphology, cytology, and immune cell infiltration in the swim bladder. **(A)** Morphological changes in swim bladder with SARS-CoV-2 spike-protein induction, and treatment with Dexamethasone and Giloy Ghanvati (GG) at different concentrations (6, 28, and 142 μg/kg/day) at the 4-days time point. Images depict lateral view with the anterior lobe (AL) and posterior lobe (PL) marked. Images were taken at ×1 magnification. **(B)**. Hematoxylin-Eosin stained cytology smears of whole swim bladder. Images were captured at ×20 objective magnification. Normal cell nuclei depicted by black arrowheads; E- Epithelial cells, and S- Smooth muscle cells. Green arrowheads show granulocytes and the yellow arrowheads show lymphocytes. **(C)** Quantitative estimation of immune cell infiltrates in the swim bladder. Data points are depicted as mean ± S.D. analyzed by Two-Way ANOVA followed by Tukey’s multiple comparisons test. *n* = 24, (####) *p* < 0.0001 compared to normal control, (****) *p* < 0.0001 compared to disease control.

At the 4-days time point, the anterior lobe (AL) and the posterior (PL) of the swim bladder in both the normal control and the humanized zebrafish control (HZF) groups showed normal appearance in size, shape, and color with continuous tunica externa and mesothelial lining of smooth muscle (Figure 3A). The disease control group (HZF-SARS-CoV-2-S) in contrast, had an inflated anterior lobe in comparison to the posterior lobe suggesting an accumulation of edematous gas. Treatment with Dexamethasone, showed the reversal of the edematous appearance and restoration of the size and shape to that seen in the normal control group. Treatment with the lowest concentration (6 μg/kg/day) of GG powdered tablet was not enough to rescue the phenotypic changes seen with the induction using SARS-CoV-2 spike protein. The anterior lobe was inflated with a discontinuous tunica externa and mesothelial lining of smooth muscle. Both the higher dilutions (28 and 142 μg/kg/day) in contrast, showed restoration of the swim bladder morphology to near normal condition as seen in the normal control group.

Cytological smears were prepared from whole mounts of the swim bladder and stained with Hematoxylin-Eosin to determine the different cell types present in the swim bladder and to quantitate the immune cell infiltration in response to the SARS-CoV-2 spike protein. The different cell types present were identified as squamous epithelium with spherical nuclei, columnar epithelium with oval nuclei, and smooth muscle cells with elongated nuclei and densely staining eosinophilic collagen. The normal control and HZF group showed normal distribution of epithelial (E, denoted by black arrowhead) and smooth muscle cells (S) as shown in Figure 3B. The disease control group showed an increase in the number of immune cells inside the swim bladder, as shown in a representative cytology image showing the presence of granulocytes (green arrowhead). Quantitative estimation of immune cell infiltrates showed a significantly higher (*p* < 0.0001) number of macrophages and granulocytes in the SARS-CoV-2 spike-protein induction models compared to the normal control (Figure 1C). Lymphocytes were conspicuously absent in the disease control model.

Treatment with Dexamethasone at 80 ng/kg/day significantly reduced the number of granulocytes with undetectable macrophage count. Conversely, the number of lymphocytes was significantly higher (*p* < 0.0001) suggesting a protective effect of dexamethasone. GG at a lower concentration of 0.2X relative to the human dose, showed a significantly lower number of granulocytes and an absence of macrophages when compared to the disease control. The number of lymphocytes was significantly higher (*p* < 0.0001) in comparison to both the disease control as well as the group treated with Dexamethasone. A dose dependent decrease in the number of granulocytes was observed at the 4 days time point, with increase in the concentration of GG given. Similarly, a dose dependent increase in the number of lymphocytes was also observed.

**FIGURE 4:**
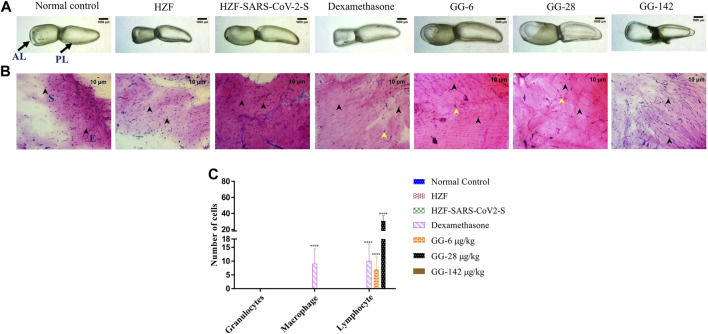
Comparison of changes in swim bladder morphology, cytology, and immune cell infiltrates at 7-days time point. **(A)** Morphological changes in swim bladder with SARS-CoV-2 spike-protein induction, and treatment with Dexamethasone and Giloy Ghanvati (GG) at different concentrations (6, 28, and 142 μg/kg/day) compared to the normal control and humanized zebrafish. Images depict lateral view with the anterior lobe (AL) and posterior lobe (PL) marked. Images were taken at ×1 magnification. **(B)**. Hematoxylin-Eosin stained cytology smears of whole swim bladder captured using ×20 objective magnification. Normal cell nuclei depicted by black arrowheads; E- Epithelial cells, and S- Smooth muscle cells. Yellow arrowheads in the images show lymphocytes. **(C)** Quantitative estimation of immune cell infiltrates in the swim bladder. Data points are depicted as mean ± S.D. analyzed by Two-Way ANOVA followed by Tukey’s multiple comparisons test. (****) *p* < 0.0001 compared to disease control, *n* = 24.

At the 7 days time point, the swim bladder dissected from the disease control group, showed much higher levels of edema with even a few of the swim bladders collapsing structurally (Figure 4A). The representative bladder showed in the figure, had swollen lobes and a collapsed ductus communicans due to an increase in pressure within the lobes. Treatment with Dexamethasone reversed some of the edematous gas accumulation but not completely as could be seen in the collapsed anterior lobe of the swim bladder. The GG treated groups on the other hand showed a reversal of the edematous appearance and the swim bladder morphology was comparable to the normal and HZF control groups.

Cytological examination of the swim bladder showed normal distribution of the smooth muscle and epithelial cells in both the normal control and the HZF group (Figure 4B). The HZF group also showed the presence of the transplanted A549 cells in addition to the normal swim bladder cells. Surprisingly, the disease control group also did not show the presence of any immune cell infiltration at the 7-days time point. This could be a result of the disease being self-limiting after several days of induction with the spike protein. Dexamethasone treatment showed a significantly higher (*p* < 0.0001) presence of macrophages and lymphocytes in comparison to the disease control group (Figure 4C). Macrophages could not be detected in any of the 3 GG treatment groups, while a significant increase in the number of lymphocytes was detected upon treatment with 6 and 28 μg/kg/day. In contrast, the highest concentration (142 μg/kg/day) of GG did not show the presence of lymphocytes.

### Recovery of Renal Damage Due to SARS-CoV-2 Spike-Protein by Giloy Ghanvati

The kidney is attached to the parenchymal layer in the dorsal wall of the body cavity. Three distinct regions could be identified in the kidney called the head, trunk (or saddle), and tail, extending from the anterior to the posterior of the body. The head of the kidney is packed with the mesonephric nephrons with glomerular tufts followed by proximal and distal tubules segments. The mesonephros (middle kidney) appears highly pigmented with melanocytes that are distributed throughout the kidney from head to tail.

**FIGURE 5:**
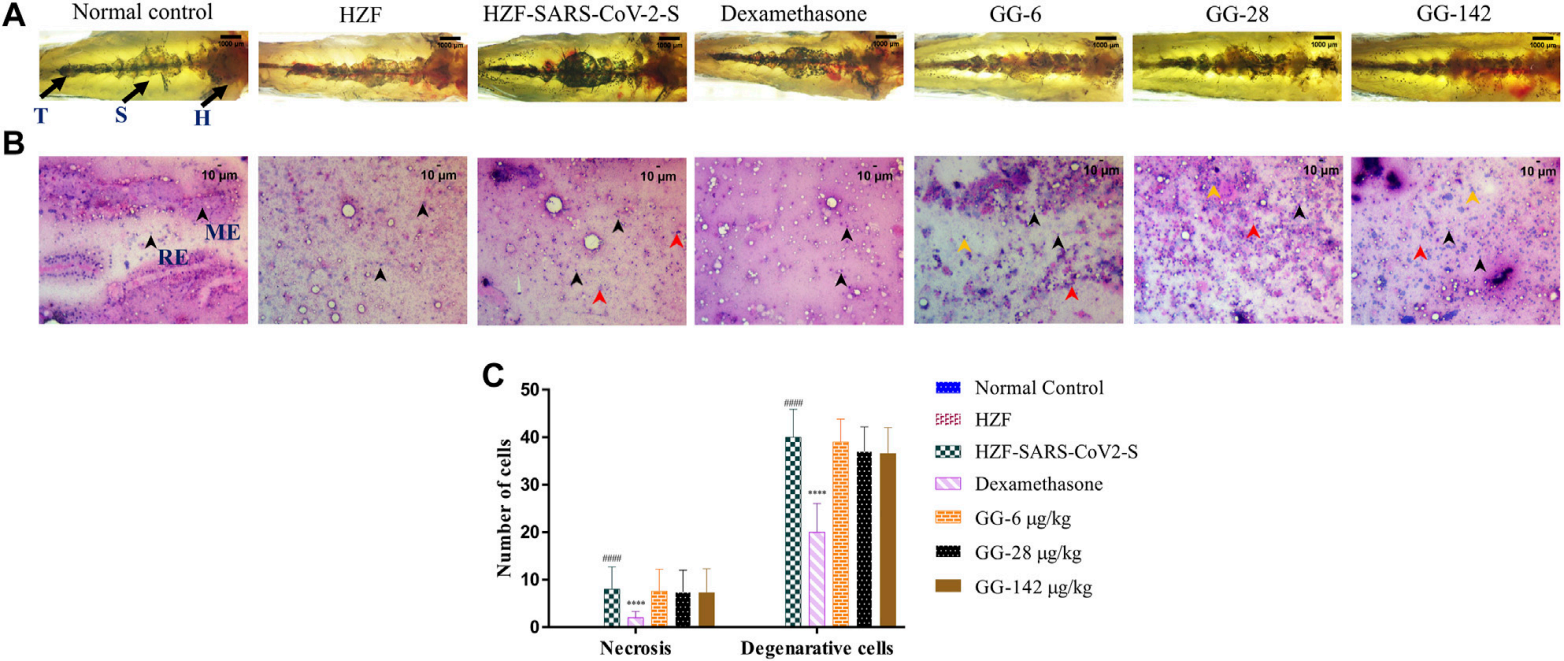
Changes to the structure, cytology, and quantitation of necrotic and degenerative cells at 4-days time point. **(A)** Structural examination of the kidney at 1X using transmitted light. Melanocytes appear as dark pigmented granules. The three regions of the kidney Head (H), Trunk or Saddle (S), and Tail (T) extend from the anterior to the posterior of the body. **(B)** Cytological examination of hematoxylin-eosin stained smears of kidney tissue imaged at ×20 objective magnification. Black arrowheads depict nuclei of normal cells, yellow show degenerative cells while red show necrotic cells. **(C)** Quantitation of kidney damage as assessed by the presence of degenerative and necrotic cells in cytological smears. Data points represent mean ± S.D. analyzed by Two-Way ANOVA followed by Tukey’s multiple comparisons test. (****) *p* < 0.0001 compared to disease control, n = 24.

The kidney in both the normal control and the HZF groups showed normal morphology with well-defined internal arrangements (Figure 5A). The Spike-protein induction group showed a loss of tubular segments and increased vascular degeneration, and a slightly larger trunk suggesting inflammation accompanied by increased necrosis. Treatment with Dexamethasone showed a densely packed arborized kidney architecture and an overall appearance similar to the kidney seen in the normal control group. Treatment with GG at all three different concentrations did not show phenotype rescue at the 4-days time point. A loss in melanocyte pigmentation and disorganized tubular segments were seen with an increase in necrosis was observed in the GG treatment groups which suggested that there was no recovery from disease phenotype at this point.

Cytological observation showed that the renal tissue from both normal control and HZF groups, had a normal pathology with evenly distributed glomerulocytes. Hematoxylin and Eosin staining revealed arborized tubular network with epithelial and mesangial cells (labeled with black arrowheads in the image) clearly visible throughout the section (Figure 5B). The disease control group in contrast, showed a disorganized cellular arrangements reminiscent of tubular necrosis. This was identified by increased erythrocyte aggregation, renal epithelial cells that are mostly basophilic with mildly stained nucleus suggesting increased number of degenerative (Yellow arrowheads) and necrotic cells (marked by red arrowheads). Treatment with Dexamethasone showed a very low distribution of necrotic and degenerative cells, and a normal distribution of epithelial podocytes. Quantitative estimation of the number of necrotic and degenerative cells showed a significant reduction (*p* < 0.0001) compared to the disease control group (Figure 5C).

At the 4 days time point, all the three doses of GG showed the presence of degenerative and necrotic cells. The quantitative estimation showed a slight reduction in the number of the respective cells in comparison to the disease control but this was not statistically significant. There was a dose dependent reduction to a very limited extent suggesting that treatment with GG did indeed have a rescue effect but perhaps to a limited extent.

**FIGURE 6:**
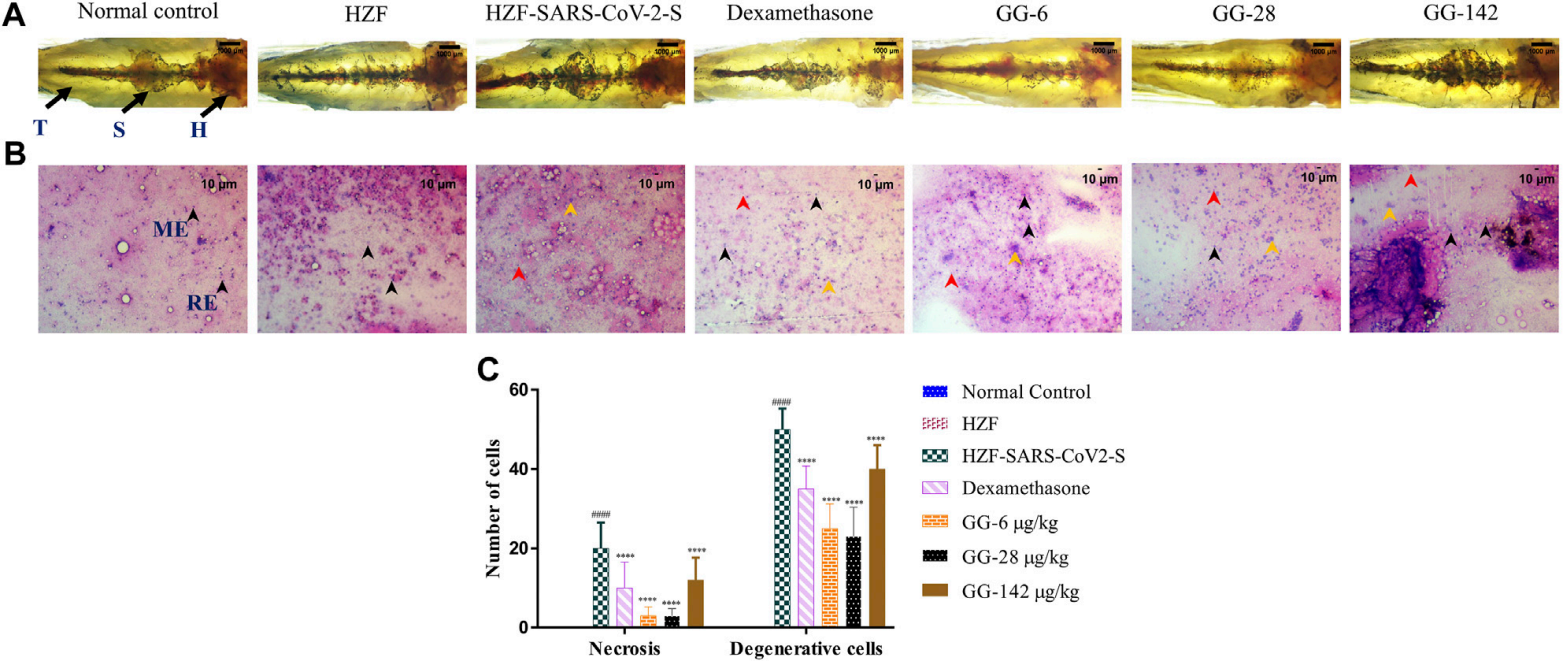
7-days time point showing changes to kidney morphology, cytology, and quantitative analysis of damage. **(A)** Structural examination of the kidney at 1X using transmitted light. Granular appearance shows the presence of melanocytes. **(B)** Cytological examination of hematoxylin-eosin stained smears of kidney tissue imaged at ×20 objective magnification. Black arrowheads depict nuclei of normal cells, yellow show degenerative cells while red show necrotic cells. **(C)** Quantitation of kidney damage as assessed by the presence of degenerative and necrotic cells in cytological smears. Data points represent mean ± S.D. analyzed by Two-Way ANOVA followed by Tukey’s multiple comparisons test (****) *p* < 0.0001 compared to disease control, *n* = 24.

Kidney morphology at 7 days time point showed normal appearance in both the normal control and the HZF control group. The kidney dissected from the disease control group showed a loss of tubular segments and increased degeneration of the kidney tissue compared to the normal control group (Figure 6A). The Dexamethasone treatment group showed structural anomalies with a disorganized glomerular tufts and melanocyte pigmentation. Treatment with GG also did not show a complete recovery from the damage induced by the presence of the SARS-CoV-2 spike protein, seen in the decreased melanocyte pigmentation and highly disorganized tubular architecture.

Cytological examination of the kidney from the disease control group (7 days time point) revealed a higher presence of degenerative and necrotic renal epithelial cells that have a mildly stained nucleus and a basophilic cytoplasm (Figure 6B). Dexamethasone treatment group showed a moderate distribution of necrotic and degenerative cells. The numbers of these cells were significantly lower (*p* < 0.0001) than that seen in the disease control group (Figure 6C). The GG treatment groups showed a gradual rescue of cytological features, wherein the lower concentrations (6 and 28 μg/kg/day) showed a dose dependent reduction in the number of both the degenerative and necrotic cells. This reduction was statistically significant compared to the disease control group. The highest concentration of GG (142 μg/kg/day) however, showed an increase in the damage to the kidney architecture with disorganized renal epithelial cells. Though there was an increase in the number of degenerative and necrotic cells in this group compared to the lower concentrations, it was still significantly lower (*p* < 0.0001) than that of the disease control group (Figure 6C). The number of necrotic cells was comparable in both the Dexamethasone and the highest GG concentration groups.

### SARS-CoV-2 Spike-Protein Induced Skin Hemorrhage Was Ameliorated By Treatment With Giloy Ghanvati

**FIGURE 7:**
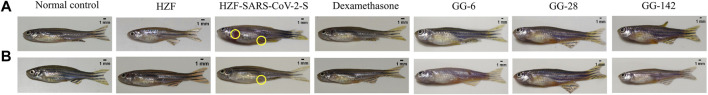
Whole body images were captured under reflected light without any magnification. Regions of skin hemorrhage are marked with yellow circles. **(A)** 4-days time point, **(B)** 7-days time point.

Skin hemorrhage, one of the clinical signs of vascular dysfunction, was not detectable in both the normal control and the HZF groups. Representative image from the disease control group at the 4 days time point, showed the presence of hemorrhagic regions near the pectoral and anal fins (Figure 7A). All the fish in this group were detected with skin hemorrhage in one of the regions near the dorsal, pectoral, anal or caudal fin. Treatment with Dexamethasone and three different concentrations of Giloy Ghanvati reversed the skin hemorrhage and the fish showed unblemished skin under reflected light.

Representative image of the fish from the disease control group at the 7-days time point showed the presence of skin hemorrhage at the anal fin region (Figure 7B). The different treatment groups with either Dexamethasone or GG showed a complete reversal of the clinical signs of skin hemorrhage as shown by the representative images.

### Treatment with Giloy Ghanvati Reversed the Abnormal Behavioral Fever Induced by SARS-CoV-2 Spike-Protein

**FIGURE 8:**
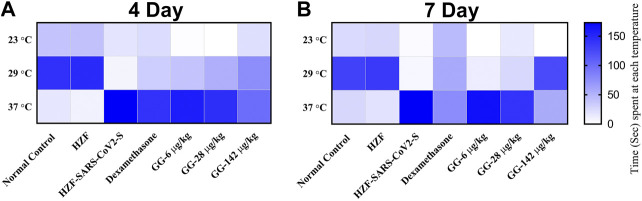
Heat map showing changes to body temperature of zebrafish as assessed by behavioral fever. The mean time spent by individual fish in each of the three temperature chambers (23, 29, and 37°C), is shown. Increase in color intensity shows longer time spent at a particular temperature.

Behavioral fever is a measure of the internal body temperature of the fish and is determined by the time spent (in seconds) at three different temperatures 23, 29, and 37°C. At the 3 days time point, the normal control and HZF group fish spent a larger part of the recorded duration at 29°C which is the normal physiological temperature (Figure 8A). Disease control group were observed to spend most of their time at 37°C indicating a change in their physiological body temperature suggestive of behavioral fever. The mean time recorded by this group at the higher temperature was significantly higher than that seen in either the normal control or the HZF group (Table 2). The fish from Dexamethasone treatment group spent most of their time at 37°C but the average time was significantly less (*p* < 0.0001) compared to the disease control group. The time spent at 29°C was also comparatively higher than the disease control group and was statistically significant.

**TABLE 2:** Changes to body temperature as assessed by behavioral fever. Time spent (in seconds) by individual fish in three different temperature chambers (23, 29, and 37°C). Data is shown as mean ± S.D. of 24 individuals, analyzed by Two-Way ANOVA followed by Tukey’s multiple comparison test (#) denotes *p* < 0.0001 compared to the normal control (*) denotes *p* < 0.0001 compared to disease control, and (a) denotes *p* < 0.0001 when compared to 37°C.

Study groups	4^th^ day time point			7^th^ day time point		
	23°C	29°C	37°C	23°C	29°C	37°C
Normal control	35.58 ± 3.0	129.63 ± 1.4	14.92 ± 2.7	24.13 ± 0.7	130.00 ± 0.8	25.88 ± 0.7
HZF	37.79 ± 1.5	134.00 ± 1.5	8.21 ± 2.0	25.88 ± 1.3	135.75 ± 1.5	18.38 ± 1.3
HZF-SARS-CoV-2	15.96 ± 1.6^#^	7.04 ± 1.3^#^	157.08 ± 1.6^#^	2.38 ± 1.1^#^	4.75 ± 1.3^#^	172.88 ± 1.3^#^
Dexamethasone	21.13 ± 2.3^*^	30.46 ± 2.3^*^	128.29 ± 1.9^*^	45.54 ± 1.6^*^	57.54 ±2.0^*^	76.92 ± 2.1^*^
GG-6 µg/kg	1.21 ± 2.1^*^	36.17 ± 2.6^*^	142.63 ± 2.3^*^	0.00^*^	11.92 ± 1.9^*^	168.08 ±1.9^*^
GG-28 μg/kg	0.58 ± 1.2^*^	48.46 ± 1.9^*^	130.96 ± 2.1^*^	14.25 ± 1.8^*^	25.13 ± 1.8^*^	140.63 ± 1.9^*^
GG-142 μg/kg	20.17 ± 4.5^*^	69.92 ± 3.5^*^	89.92 ± 4.2^*^	0.00^*^	123.96 ±2.0^*/a^	56.04 ± 2.0^*^

The average time spent by all the 3 GG treatment groups at 37°C, was higher than the time spent at 29°C, respectively. However, when compared to the disease control group, the time spent by each of the different treatment groups at 37°C was significantly lower (*p* < 0.0001). There was a dose dependent increase in the time spent at 29°C when treated with GG.

Fish from the Dexamethasone group, at the 7-days time point, showed comparable time spent at all the three temperature chambers (Figure 8B; Table 2). This suggests that this group of fish is recovering from behavioral fever. Treatment with low doses of GG (6 and 28 μg/kg/day) did not have much effect on the behavioral fever as the fish spent more time at 37°C compared to 29°C. However, the average time spent by these groups at 37°C was significantly lower than that of the disease control group. GG at 142 μg/kg/day was able to reverse the behavioral fever phenotype as the fish spent significantly more (*p* < 0.0001) time at 29°C compared to 37°C (Table 2).

### Giloy Ghanvati Reversed SARS-CoV-2 Spike-Protein Induced Mortality

**FIGURE 9:**
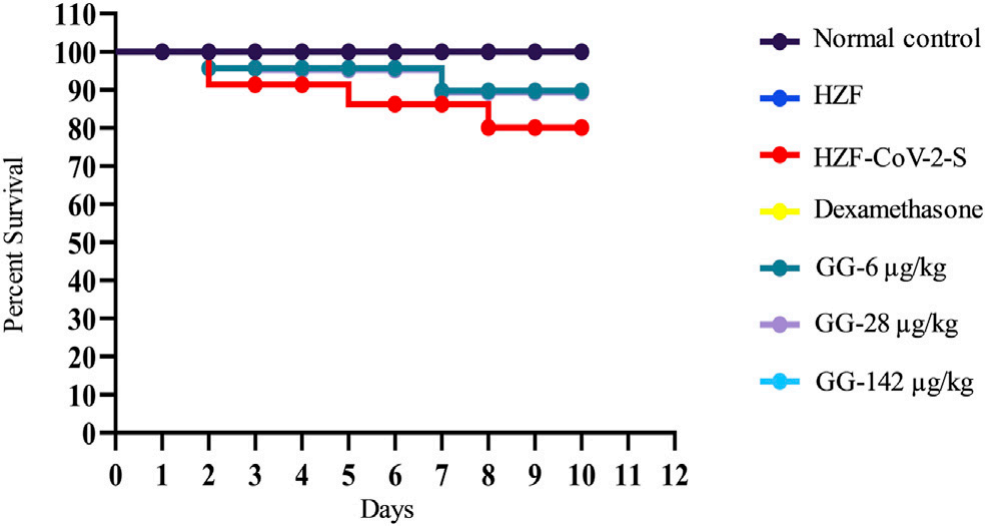
Mortality in the different study groups was recorded every day for 10 days in a parallel experiment. Survival analysis is presented as Kaplan-Meier curve.

Mortality in the study groups was recorded daily and the percent survival was calculated in a parallel experiment which was observed for 10 days. As expected the normal control and the HZF group showed 100% survival, whereas the disease control group had only 80% survival (Figure 9). Treatment with Dexamethasone restored the survival of that group comparable to the normal control. In the GG treatment groups, 100% survival was seen only with the highest concentration, whereas both 6 and 28 μg/kg/day showed 89% survival.

## Discussion

In this study, we used a formulation tablet named Giloy Ghanvati (GG) which is prepared from an aqueous extract from *Tinospora cordifolia* (willd.) Hook. f. and Thomson. *T. cordifolia* belongs to a group of medicinal plants used for the treatment of many different conditions including cold and fever (Chi et al., 2016). Cordifolioside A was reported to have immune-modulatory effects and an increase in the phagocytic activity of neutrophils was attributed to the synergistic effect of various phyto-compounds isolated from *T. cordifolia* (Giloy) (Sharma et al., 2012). In our study we identified Cordifolioside A, Palmatine, *β*-Ecdysone, and Magnoflorine from GG, in HPLC analysis.

Magnoflorine is a member of the aprophine derivatives of isoquinoline alkaloids, and is very widely distributed in the plant kingdom. The compound has been shown to have a widely varying pharmacological effects including anti-inflammatory and immunomodulatory activities (Sharma et al., 2012; Wang et al., 2019). Though the mode of action is not fully known, *in vitro* and *in vivo* studies showed improved phagocytic activity of macrophages (Ahmad et al., 2016; Ahmad et al., 2018). Cordifolioside A is a phenylpropene disaccharide and has been shown to have immune-stimulatory activity in mice challenged with sheep RBCs, as measured by antibody production (Maurya et al., 1996). Macrophage cell line when treated with Cordifolioside A showed increased activity of NADH-oxidase, NADPH-oxidase and myeloperoxidase, all three of which are signs of macrophage activation (More and Pai, 2012).

Beta-Ecdysone and other ecdysteroids appear in many plants as protection agents against herbivorous insects and are purported to have medicinal value (Dinan et al., 2001). Phytoecdysteoids have been identified in more than 100 plant families and plants are capable of biosynthesis of these from mevalonic acid, unlike insects (Adler and Grebenok, 1995). The most commonly found phytoecdysteroid is 20-hydroxyecdysone (beta-ecdysone) and has also been identified in *T. cordifolia* (Abiramasundari et al., 2018) and has been demonstrated to induce osteogenic transformation. They have been shown to have immunomodulatory activity wherein the concentration of antibody forming B cells in the spleen of mice immunized with sheep RBC was higher (Sakhibov et al., 1989). Recent studies by LaFont et al. (LaFont et al., 2020) suggest a beneficial effect of ecdysone on the Renin-Angiotensin system. This could be especially beneficial in the SARS-CoV-2 infection as the ACE-2 receptor is also involved in the proper maintenance of the Renin-Angiotensin System.

The final compound detected in our study was Palmatine, an isoquinoline alkaloid, that is present in a wide variety of plants (Tarabasz and Kukula-Koch, 2020). The anti-inflammatory activity of this compound may be due to its activity in suppressing COX-2 and also as a prostaglandin biosynthesis suppressor (Küpeli et al., 2002). It has also been shown to inhibit Respiratory Syncytial Virus (RSV) (Lee et al., 2017), through the induction of Type 1 Interferon and its associated signaling pathways. It was also found to be prophylactic in mice.

The xeno-transplant model was used to replicate the human responses by introducing A549 cells into the swim bladder. The fish were observed for a period of 7 days and no changes in the normal behavior of the xeno-transplanted fish. During the experimental period, behavioral fever was used as a measure of fever associated with inflammation or an immune response to the presence of foreign cells and the lack of any changes suggested that there was no adverse effect due to the presence of these cells. Studies by Shen et al. (Shen et al., 2020) showed that the A549 cells colonize well even within the larvae of zebrafish and do not cause any untoward developmental changes in the larvae.

In our study, we identified increased infiltration of granulocytes and macrophages in swim bladder of the SARS-CoV-2 spike-protein induction group. There was a significant reduction in the number of granulocytes in all the 3GG treatment groups (6, 28, and 142 μg/kg/day), and showed dose dependency with an increase in concentration. Surprisingly, macrophages were not detected in either the Dexamethasone or GG treatment groups. Studies in rats (Koppada et al., 2009), were able to show that *T. cordifolia* extract was able to not only downregulate the production of various inflammatory mediators but also activate lymphocytes and increased humoral response in Mozambique *Tilapia* (Sudhakaran et al., 2006). It can be speculated that the decreased number of macrophages in the GG treatment groups could be a result of increased T cell and B cell response than macrophages. This could be corroborated by the significant dose dependent increase in the number of lymphocytes seen in the GG treatment groups.

The production of pro-inflammatory milieu at the site of SARS-CoV-2 spike-protein introduction was reflected in the morphological changes in the swim bladder. The bladder was inflamed and in some cases structurally collapsed due to edema, which is a response to the presence of pro-inflammatory cytokines and immune cells. This change in the morphology was not fully recovered upon treatment with Dexamethasone even at the 7-days time point. Treatment with GG at two different concentrations (28 and 142 μg/kg/day) was able to reverse the swim bladder changes at the 4-days time point itself. This suggested that GG is very effective in reducing the pro-inflammatory milieu seen in the presence of SARS-CoV-2 spike protein. These results are in line with our previous results using a tri-herbal formulation containing *T. cordifolia* ((Balkrishna et al., 2020).

The reduction in the pro-inflammatory cell infiltrates can be attributed to the reduction in the pro-inflammatory cytokines by GG. Studies have shown that Magnoflorine reduced LPS induced expression of TNF-α, IL-6, and IL-1β, in mice, in a dose dependent manner (Guo et al., 2018). In addition, Palmatine was also shown to inhibit these cytokines in Endometrial Epithelial Cells (gEEG) (Yan et al., 2017). These studies provide a mechanistic insight into the action of GG in reducing pro-inflammatory responses seen with the SARS-CoV-2 spike protein.

The “cytokine storm” syndrome is known to cause systemic shock and multi-organ failure in severe cases, when left untreated (Mokhtari et al., 2020). Acute kidney injury was seen in about 29% of the severe cases. ACE2 receptor which is needed for successful viral entry into cells is expressed on almost all the cell types seen in the kidney such as the proximal tubule epithelium, endothelium of glomerulus, and podocytes (Gheblawi et al., 2020). The Renin-Angiotensin system which plays a key role in maintaining proper kidney function is known to be activated via the ACE2 receptor and interference in this due to SARS-CoV-2 binding to the ACE2 receptor can induce injury to the resident renal cells (Ingraham et al., 2020).

Loss of tubular segments and vascular degeneration was observed in the disease control and was much more severe in the 7-days time point compared to the 4-days time point. Treatment with GG reduced the renal cell necrosis and the number of degenerative cells in a dose dependent manner at the 4-days time point. However, treatment with 142 μg/kg/day GG showed higher number of necrotic and degenerative cells at the 7-days time point compared to the lower doses at the same point. The number of either type of cells was comparable to the Dexamethasone treatment group and was significantly lower than that seen in the disease control group. Ethanolic extracts of *T. cordifolia* containing Palmatine, in addition to other phyto-compounds, showed protection against nephrotoxicity induced by either aflatoxin-b (1) (Sharma and Gupta, 2011) or gentamicin (Khaksari et al., 2019). It has been shown to have these protective effects due to its powerful anti-oxidant activity.

Apart from the gross cytological and morphological changes seen in various organs of the disease control fish, the disease control showed a clear preference for water at 37°C. This behavior fever is a reflection of the body temperature and showed that the disease control fish had a high fever. Treatment with Dexamethasone for did not reverse the behavioral fever at the 4-days time point while there was a trend toward normalization at the 7-days time point. Lower concentrations of GG also did not reverse the behavioral fever even at the 7-days time point but treatment with 142 μg/kg/day totally reversed the behavioral fever showing that the highest dose given for 6 days (7-days time point) was able to rescue the SARS-CoV-2 spike-protein induced disease phenotype. The mortality seen with SARS-CoV-2 spike-protein was completely reversed with the highest dose of GG but lower doses (6 and 28 μg/kg/day) could only rescue mortality to 89% over a period of 10 days.

## Conclusion

The present study shows the pharmacological effects of the aqueous extracts of *Tinospora cordifolia* (willd.) Hook. f. and Thomson in the form of Giloy Ghanvati (GG) tablets against SARS-CoV-2 spike-protein induced disease phenotype in a humanized zebrafish model. Treatment with GG reversed the pro-inflammatory cell infiltration in the swim bladder and also rescued the tubule damage and necrosis seen in the kidney. SARS-CoV-2 spike-protein induced fish demonstrated behavioral fever indicative of higher body temperature and this was reversed upon treatment with the test formulation. Taken together, the morphological, cytological, and behavioral changes observed with induction of SARS-CoV-2 spike-protein were rescued to near normal levels with GG treatment.

## Data Availability

The raw data supporting the conclusion of this article will be made available to the scientific community upon request.
